# Dyeing wool fabrics with specialized dyes and their mixtures using supercritical CO_2_

**DOI:** 10.1038/s41598-025-25134-w

**Published:** 2025-11-18

**Authors:** Hanan Elsisi, Tarek Abou Elmaaty, Elham Negm, Shahinaz Abouelenin

**Affiliations:** 1 Department of Textile Printing, Dyeing and Finishing, Faculty of Applied Arts, Damietta University, New Damietta, 34512 Egypt; 2 Chemistry Department, Faculty of Science, Damietta University, New Damietta, 34517 Egypt

**Keywords:** Supercritical carbon dioxide, Wool fabrics, Color matching, Disperse reactive dyes, Chemistry, Environmental sciences, Materials science

## Abstract

Wool has been colored in supercritical carbon dioxide in a few trials, yielding only a small spectrum of colors, mostly orange and yellow, with minimal research on alternative color combinations. To further the industrial development of supercritical carbon dioxide (SCCO_2_) dyeing, a variety of dyes must be used to produce a diversity of colors. The material’s overall appearance is affected by shades of blue, yellow, and red. This study revealed the generation of new colors using three reactive disperse dyes with various colors: a blue dye derived from the anthraquinone parent body, a yellow dye with a pyrazole moiety, and a third, red, dye with an azo-thiazole moiety. These dyes and their blends were employed to dye wool fabric using supercritical carbon dioxide. The maximum *K/S* values were attained at 11.14 for the mixture of (blue dye: red dye 80:20), while the lowest *K/S* was indicated at 8.43 for (blue dye: red dye: yellow dye 1:1:2). However, the compatibility grade as per RCR values showed excellent values for the combination of three dyes. The dyed samples were evaluated for colorfastness, and the results showed that they retained their color well and were exceptionally durable after washing. The growing patterns in both the dyeing rate and build-up curves indicate good compatibility. Furthermore, desired hues of Teal, green, olive, gray, and reddish-brown can be created by combining blue, red, and yellow dyes in varied ratios in supercritical CO_2_.

## Introduction

Polluted ecosystems and diminishing natural resources have raised considerable and severe concerns on a global scale since the turn of the twenty-first century^1,2^. The textile dyeing and finishing industry faces substantial barriers to long-term sustainability, primarily due to environmental issues such as the lack of water resources, severe water damage from facilities, and the challenges associated with wastewater treatment^3,4^. Traditional dyeing or finishing procedures are a significant contributor to this matter, as they require a considerable amount of water, more than 100–150 L per kilogram of fiber, and 8–18 MJ of energy^5,6^. The global production of organic dyes and pigments is estimated to be over 7 × 10^5^ tons, with 10–15% of it discharged into wastewater, which constitutes a significant threat^4^. Therefore, manufacturing functional and environmentally sustainable products, as well as adopting cost-effective and emission-reducing procedures, are crucial for the growth of the contemporary textile manufacturing sector^7^.

The supercritical carbon dioxide dyeing (SCDD) method is not just a potential solution, but a promising one, to the environmental challenges faced by the textile industry. It is regarded as an eco-friendly dyeing process compared to traditional dyeing techniques, as supercritical carbon dioxide (SCCO_2_) is a non-hazardous, eco-friendly medium with lower viscosity and higher diffusivity than water-based media, and is recyclable^8–10^. It has greater potential for dye diffusion into materials during dyeing processes^11,12^. Furthermore, the unattached dye, alongside other remaining chemical reagents, can be successfully separated and removed by simply lowering the system pressure.

Compared to typical water dyeing, SCDD can mitigate economic and environmental drawbacks, and the application of SCCO_2_ significantly reduces the consumption of both water and energy^13–16^. SCCO_2_ was first employed in laboratory coloring for textiles in 1988, and since then, it has been extensively studied and improved in the dyeing and finishing of textiles. Recently, SCCO_2_ media have been satisfactorily used to color synthetic fibers, such as nylon^17–19^, polyester^20–23^, and polypropylene^24–27^, and are undergoing pilot-scale and industrial-scale production^27^. On the other hand, conversely, the study of SCDD of natural textiles is a fast-growing field that holds great potential for the manufacture of sustainable textiles. To cover all of the aspects and fulfill its full potential, further research must be undertaken.

Wool is not just a remarkable protein fiber, but a highly versatile one that deserves to be acknowledged as one of the original natural fabrics for clothing. Its inherent qualities—including outstanding flexibility, superior heat retention, high moisture absorption, exceptional stain and static resistance, a light sheen, and easy handling—all contribute to its long-term value. Wool, therefore, has a higher ignition temperature than most conventional textiles, which enhances fire safety. These characteristics combine to position wool as a significant contributor to sustainable textile production, promoting an even more environmentally friendly world^28–30^.

The SCDD of natural materials, notably wool, remains a significant challenge. A substantial challenge involves identifying suitable dyes for this medium that effectively color natural fabrics without compromising their inherent properties^31^. This issue revolves around the fact that the SCDD of natural fibers, such as wool, is hampered by their greater polarity and crystallinity than that of synthetic fibers, which have the plasticization impact of SCCO_2_ on polymers (e.g., polyester fabric) that enhances the mobility of the polymers and boosts the migration of semi-crystalline polymers^31,32^. Compared to synthetic fibers, the non-polarity of SCCO₂ makes it mostly ineffective in swelling polar wool fibers, resulting in a lower-quality dyeing yield^33^. In this regard, the presence of amino functionalities within solute molecules is well-documented to prevent the dissolving and swelling behavior of wool in SCCO_2_^34^.

Consequently, the low affinity of disperse dye to wool fabric in SCCO_2_ prompted researchers to develop dyes^35^ and/or modify natural fibers to achieve better results for SCDD of natural fibers^36,37^.

The development of reactive disperse (RD) dyes stands out as the most prevalent strategy^38^. This method enables the dyeing of natural fibers in SCCO_2_ medium by introducing reactive groups to disperse dyes^39^. RD dyes have a limited range of applications in SCCO_2_ media, and there are specific restrictions on their use in SCCO_2_ dyeing procedures^40,41^. RD dyes are required to achieve preferred solubility in hydrophobic media while maintaining a high affinity for fabric absorption to accomplish excellent SCDD performance on natural fibers. These dyes should also include reactive groups, including hydroxyls and amines, that react rapidly with the functional groups of fibers. This method results in the covalent attachment of dye molecules to fibers, which promotes fixation rates and colorfastness^41^. Unfortunately, few studies have investigated SCDD of wool fabric with RD dyes^1,35,41–43^.

Notwithstanding, developing new colors of wool fabrics with high color strength features in SCDD remains a significant hurdle, as limited colors have been documented in prior studies, which have focused primarily on the three primary colors.

This involves the development of new color palettes for SCCO_2_-dyed wool. A vast range of colors, tones, and hues can be created by strategically combining specifically prepared dyes, which promotes the growing popularity of SCDD in textile manufacture. Although it has substantial practical significance, the compatibility of dyes in admixture with SCCO_2_ is a rather understudied issue in scientific papers. This lack of study requires further exploration to fully realize the promise of these understudied matters^44^. The compatibility of blended dyes is a crucial factor in determining their color-matching effectiveness under various dyeing conditions. This assessment lays the framework for the very efficient manufacture of color-matched fabrics^45^.

In contrast, the multi-step SCDD procedure for fabrics dramatically extends processing time and procedure complexity. This diminishes process viability owing to the subsequent increase in dyeing costs. Unfortunately, prior studies on color matching with blended dyes in SCCO_2_ concentrated primarily on synthetic fabrics, particularly polyester, with limited success in achieving high color strength^14,44,46^. However, our previous work successfully generated a range of new colors on cotton fabric using various dye mixtures in a single SCDD process, along with antibacterial properties^47^.

In this study, to complement the different color range for wool fabrics, three different dyes were used to mix and produce new colors. The first is blue, derived from the anthraquinone family, the second is yellow, incorporating a pyrazole moiety, and the third is red, derived from an azo-thiazole dye. The dyeing behaviors and capability to match the colors of three RD dyes on the wool fabric were studied using dyeing rate curves.

## Experimental

### Materials and chemicals

Bleached (100%) wool fabrics have been purchased from Golden Tex for wool in the 10th of Ramadan City, Egypt. Most of the chemical substances were obtained from Sigma-Aldrich and used directly without further purification. The solvent used for the supercritical dyeing was CO_2_ (99.6%, supplied by NETCO Industrial Company, Cairo, Egypt), and the co-solvents were gradient-grade methanol and acetone (both with 99.8% purity). The dyes used in this study (Table 1) were synthesized and described in our previous study^47,48^***.***

**Table 1:** Chemical structures of dyes.

Blue dye	1, 5-diamino-4-((4-((4, 6-dichloro-1, 3, 5-triazin-2-yl)amino)phenyl)amino)-8-hydroxyanthracene-9,10-dione
Yellow dye	N-(3-(3-chlorophenyl)-4-((4-chlorophenyl) diazenyl)-1 H-pyrazol-5-yl) ethane sulfonamide
Red dye	N-(pyridin-2-yl)-4-((4-(p-tolyl)-2-(vinylsulfonamido)thiazol-5-yl) diazenyl)benzenesulfon amide

### SCDD of the wool fabrics

The procedure for the SCDD process was determined based on our prior study^47,48^ as follows:

*Step 1* The purified dyes were placed at the bottom of the vessel and gently blended with the studied mixture and concentration. Then, (3 × 10 cm) of wool fabric was warped for dyeing in the same way as our previous study. The stainless steel vessel has a maximum internal volume of 50 ml.

*Step 2* A high-pressure valve (model JASCO BP-4340) and a CO_2_ pressure pump (model JASCO PU-4386) were used to introduce CO_2_ from the gas cylinder into the dyeing apparatus after it was chilled to a liquid using a chiller (model Julabo FL601). The highest level of pressure was 250 MPa.

*Step 3* After turning on the CO_2_ circulation system, the dyeing process started. The pressure and temperature were adjusted appropriately. The thermal compensating jacket (type HC-2068-01) maintained a steady temperature during the dyeing procedure.

*Step 4* The colored material was separated from SCCO_2_ in the separator unit. Following the allotted time, the temperature was progressively lowered. Eventually, the pressure dropped to atmospheric levels. Following that, the dyed textiles were removed from the dyeing container for further analysis.

*Step 5* The wool samples were washed with room-temperature acetone to remove any unfixed colors that had adhered to the surface of the wool fabric.

### Color study evaluation and compatibility of blended-dyed wool fabrics

Following SCDD under different circumstances, the Kubelka–Munk Eq. (1) was used to determine the value of color strength (*K/S*) in the visible wavelength range (360–740 nm):

1 $$K/S = \left( {1 - R} \right)^{2} /\left( {2R} \right)$$

In which S symbolizes the scattering coefficient, R refers to the dyed sample’s reflectance, and K indicates the absorption coefficient.

A Konica Minolta spectrophotometer (Japan; model CM-3600 A) was used for calculating five characteristics of the colored wool samples: hue angle (h*), b*, which indicates yellow/blue chromaticity coordinates, chroma, a*, which means red/green chromaticity coordinates, and L*, which symbolizes lightness/darkness. Using Eq. (2), the overall color difference (∆E) was calculated.

2 $$\Delta E_{ab}^{*} = \sqrt {(L_{2}^{*} - L_{1}^{*} )^{2} + (a_{2}^{*} - a_{1}^{*} )^{2} + (b_{2}^{*} - b_{1}^{*} )^{2} }$$

The coefficient of variation percentage (CV% %) of *K/S* values was calculated using data from 10 distinct spots on the dyed wool samples.

Equation (3) was employed to calculate dye fixation based on the amount of dye taken up by the wool following the dyeing and extraction procedures.

3 $$F = \frac{{\left( {K/S} \right){\text{ extr}}}}{{\left( {K/S} \right){\text{ dyed}}}} \times 100\%$$

The change in colors caused by variations in dyeing procedure factors, which are the primary variables responsible for the main color difference between the dyed wool and the control specimen when dyed under different conditions using varying amounts of a binary blend of dyes, is reflected in the color difference index (CDI) values.

Once various ratios of binary combinations of dyes are attached to the single fiber, the value magnitudes of the corresponding ∆E, ∆C, ∆H, and MI are utilized to determine the CDI (Color difference index), regardless of their sign or direction.

The compatibility level is indicated by a compatibility rating, which ranges from 0 to 5. The CDI index is utilized in conjunction with the relative compatibility rating (RCR) technique to assess the compatibility of three dyes in binary and ternary blends. By evaluating the variance between the highest and lowest CDI values, the corresponding compatibility technique calculates the color build-up rate^49–51^.

When dyed samples were performed under ideal circumstances, the colorfastness was assessed by applying AATCC standard techniques, including rubbing (AATCC 8–2001), washing (AATCC-61-2A-1996)^51,52^, light fastness (AATCC-16A-1972)^53^, and sweat fastness (AATCC 15–1997)^50^. The stability, durability, and rechargeability of the mixed dyes on the wool fabric towards multiple laundering cycles (up to ten washing cycles) were assessed by applying AATCC Test Method 61 (2A)-1996^48^.

### Statistical analysis

Each evaluation was based on the average of the three outcomes. By using a specific formula, the standard error of the average was computed and found to be around ± 0.1.

$${\text{SE}} = \frac{S}{\surd n}$$

where n indicates the sample size and S is the sample standard deviation^47^.

## Results and discussion

Since compatibility reveals the extent to which several colors are harmoniously combined, it is a key factor to consider when evaluating the dyeing properties of dyes. Compatibility between three dyes in a binary and a ternary combination is a significant factor for producing compound hues on textiles; hence, it is imperative to avoid employing incompatible dyes for this reason. The multiple colors in the mixture have variable degrees of color build-up, which makes it tricky to precisely manage the formation of compound hues to produce a desired color tone and shade. Compatibility between dyes is defined as having the same or almost identical exhaustion characteristics. There are several techniques for determining if two or three dyes in a binary and ternary combination are compatible. Nevertheless, handling the color shade can turn out complicated when applying dyes that have various dyeing rates because variables in SCDD such as pressures (15, 20, and 25) MPa, dyeing temperatures (90, 100, and 110) °C the percentage of dye (1 to 6%), and static times (60, 90, and 120) minutes may individually have a substantial effect on the result. On the other hand, when dyes are well-matched and compatible, creating the desired color hue is more straightforward since the dissolution, diffusion, and adsorption characteristics of the dyes in SCDD operate effectively. A blend of the investigated dyes (50–50%) was applied in single-factor studies to optimize the SCDD of wool textiles. Various combinations of blue, red, and yellow dye, ranging in ratio from (80–20)%, (72–25)%, (20–80)%, and (25–75)%, were studied under various dye concentrations and ideal circumstances.

### Dyeing rate curves for the reactive disperse dyes and their mixture on wool

#### The effect of dye concentration on dyed wool samples

**Fig. 1:**
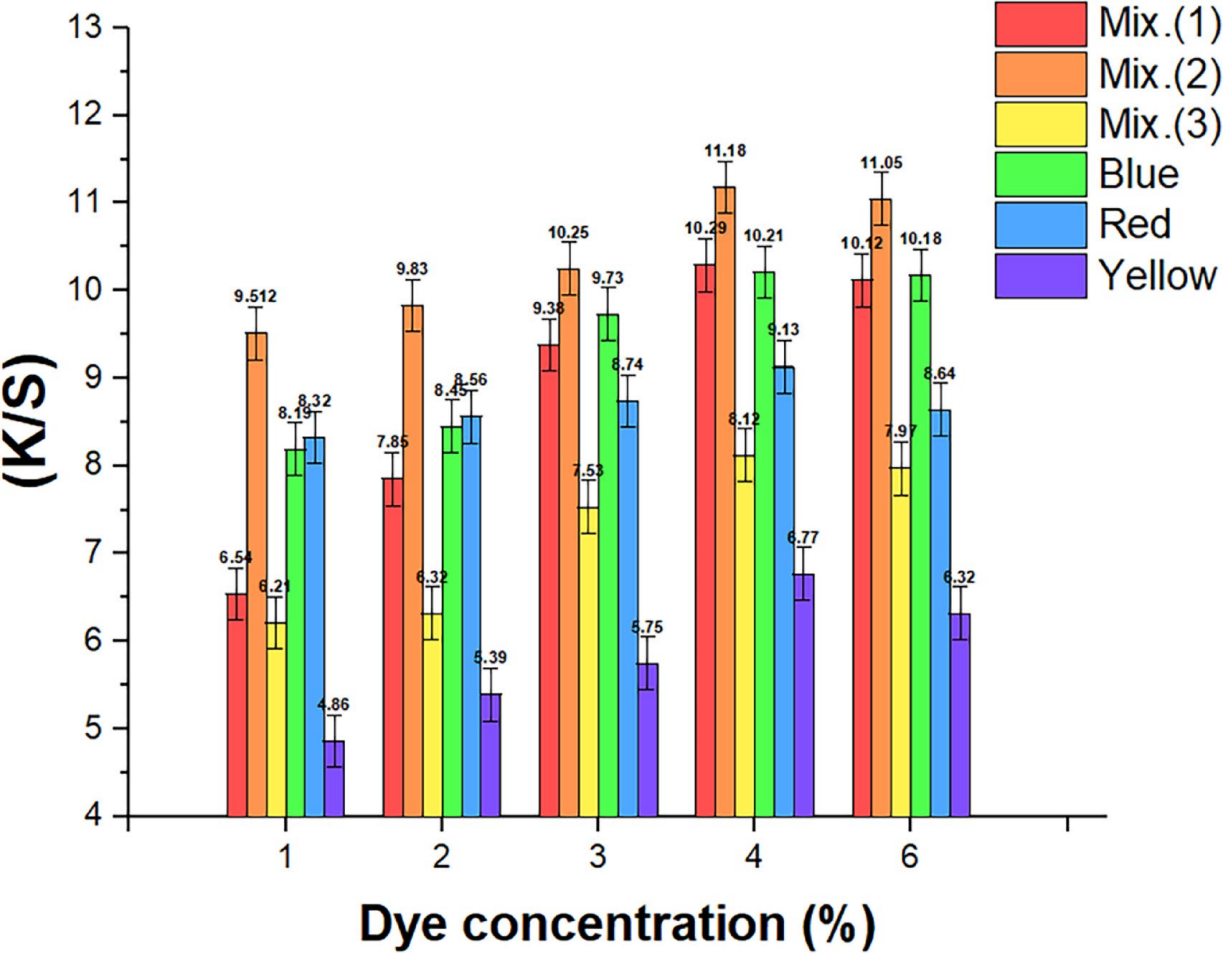
The accumulation curves for the (50:50) and 50:50:50 mixture of the three RD dyes. (Mix 1 symbolizes a mixture of yellow and blue dyes, Mix 2 symbolizes the mixture of red and blue dyes, and Mix 3 symbolizes the mixture of yellow, red, and blue dyes).

A comprehensive understanding of the coloration, dye shade, and dye binding method was obtained by observing the accumulation of three RD dyes on wool fibers. RD accumulation curves were analyzed in SCDD using dye concentrations increasing from 1 to 6% (o.w.f.). As shown in Fig. 1**,** with the increase of dye concentrations, the (*K/S)* ratings of the dyed wool improved and developed when dye concentrations ranged from (1 to 4) % (o.w.f.) for three different colors and their mixtures (Mix. 1, Mix. 2, and Mix. 3). In contrast, When the dye concentration exceeds 4% (o.w.f.), no noticeable boost is observed. As a result, the dye molecules’ driving power increased, blocking the textiles’ pores and causing the substances to get saturated to a depth of 4%. The ability to absorb dyes may be limited to 6% due to a steady slowdown in the diffusion speed of dye particles.

#### The effect of ratios of dyes mixture on dyed wool samples

**Fig. 2:**
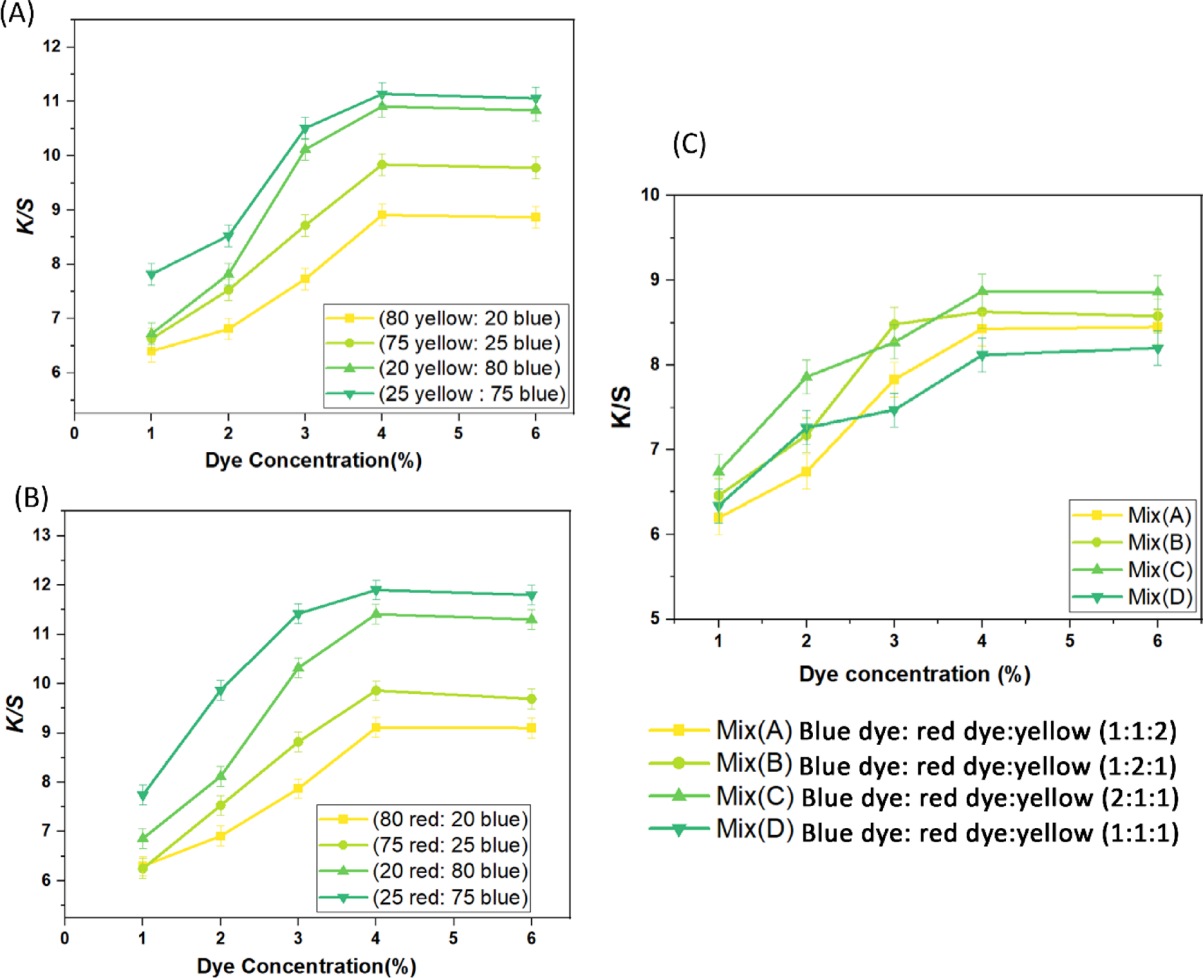
The accumulation curves for various ratios of the blends of the three RD dyes. (**A**) Different ratio of mixture of Y and B dyes, (**B**) different ratio of mixture of R and B dyes, and (**C**) different ratio of mixture of B, R, and Y dyes.

The color-matching features of the yellow, red, and blue RD dyes at different dye blending ratios and concentrations are demonstrated in Fig. 2. The findings reveal that all dye mixtures exhibit outstanding color strength build-up characteristics, as indicated by the increase in *K/S* rating with increasing dye concentrations. As seen in Fig. 2, as various mixtures of the different dyes are mixed, the *K/S* results of the wool samples progressively improved by increasing the dye concentration from (1% to 4% o.w.f. However, even though the dye concentrations went from (4% to 6) % (o.w.f.), the *K/S* readings significantly improved when various ratios of a binary and ternary combination of the selective dyes (such as 80:20, 75:25, 50:50, 20:80, and 25:75 B dye and Y dye) were applied to the wool samples. Since the yarns are already saturated by the adsorption of dye when the dye concentration reaches 4% (o.w.f.), the *K/S* readings do not rise noticeably.

#### The effect of the temperature on dyed wool samples

**Fig. 3:**
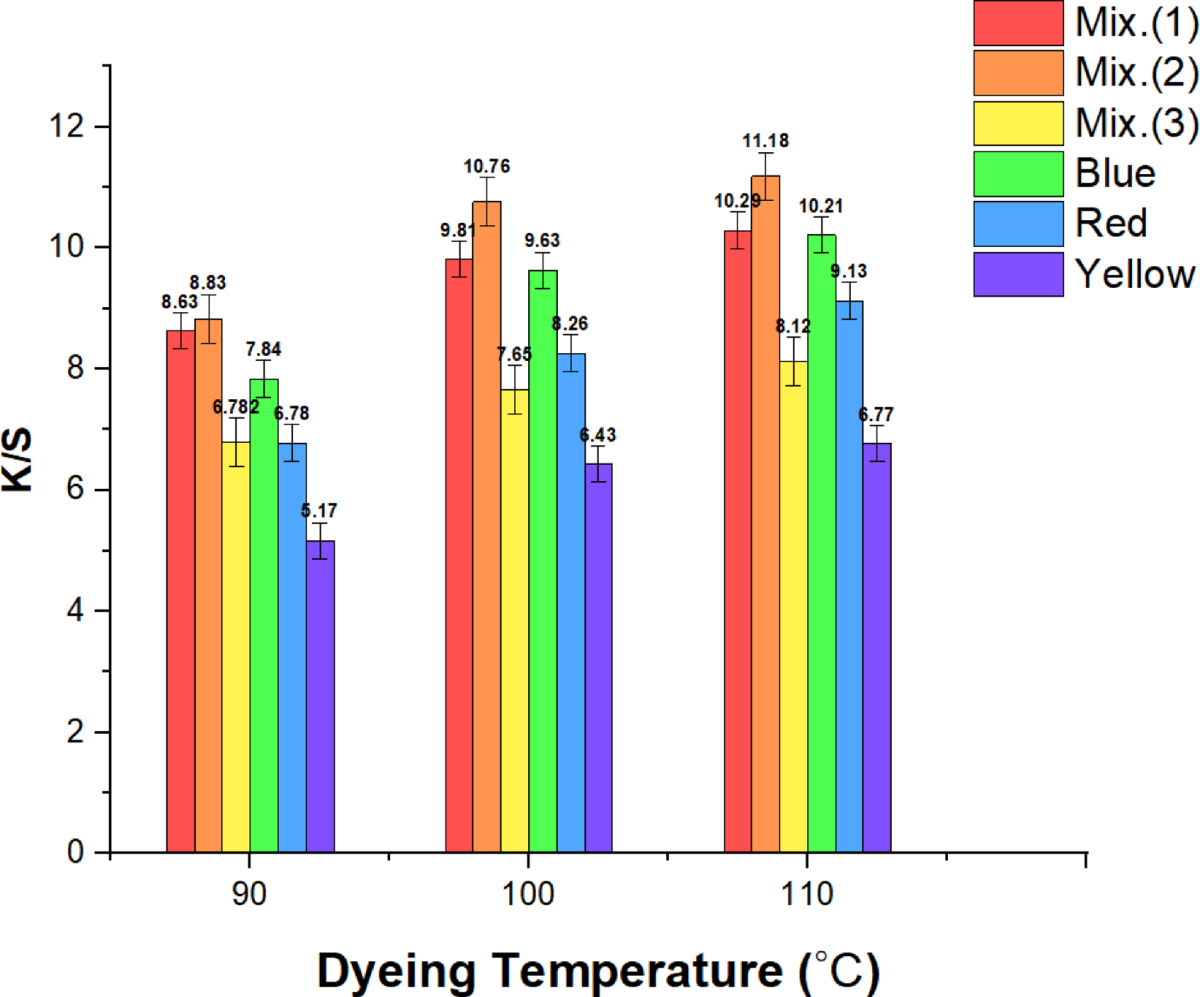
Relationship graphs for the three dyes under investigation and their 50:50 combination between dyeing temperature and *K/S*. (Mix 1 symbolizes the mixture of yellow and blue dyes, Mix 2 symbolizes the mixture of red and blue dyes, and Mix 3 symbolizes the mixture of yellow, red, and blue dyes).

Employing a 4% dye concentration, a 90-min dyeing time, 25 MPa pressure, and systemic temperatures ranging from ( 90 to 110) °C, Fig. 3 demonstrates how temperature impacts the *K/S* of dyed wool. The results indicate that when the temperature increases, the *K/S* also becomes higher. This occurs because higher temperatures allow the pigment molecules to permeate the fabric more effectively, triggering larger pores and increased diffusion due to the dye’s enhanced swelling and reactivity. The dye’s reactivity was enhanced dramatically once the temperature exceeded (90 to 110) °C, exactly as there were notable improvements in fixation, suggesting that more covalent bonds were generated as the temperature increased.

#### The effect of pressure on dyed wool samples

**Fig. 4:**
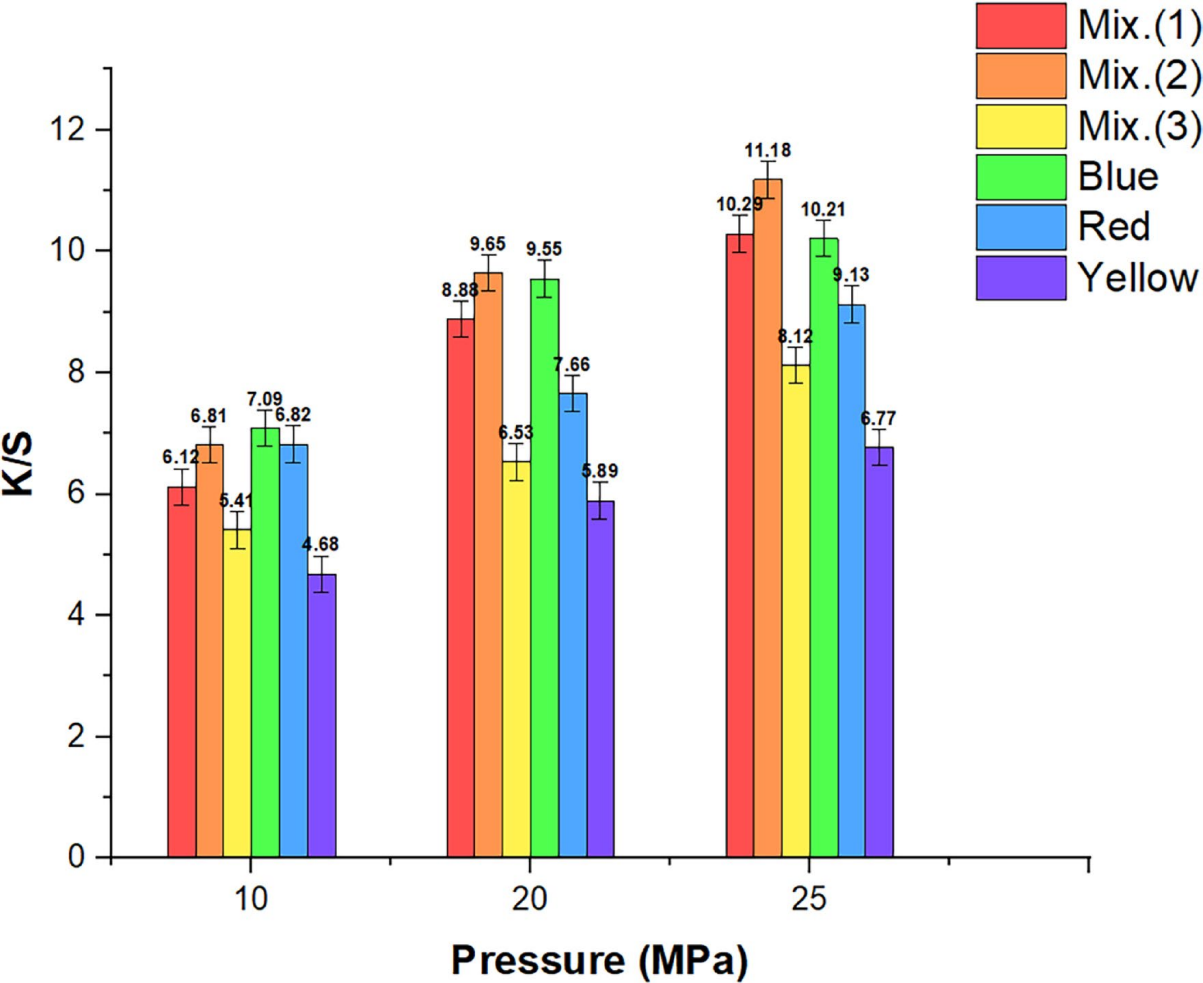
Relationship graphs for the three dyes under investigation and their 50:50 combination between dyeing pressure and *K/S*. (Mix 1 refers to the mixture of yellow and blue dyes, Mix 2 refers to the mixture of red and blue dyes, and Mix 3 refers to the mixture of yellow, red, and blue dyes).

To understand how system pressure affects the color-matching compatibilities and absorption characteristics of the three RD dyes. Three different system pressure levels were examined: (15, 20, and 25) MPa. Figure 4 displays the study’s outcomes, demonstrating the varied and substantial implications of pressures on the absorption characteristics and compatibilities of the two and three dyes under investigation. Various and overall proportional boosts in the curves highlight this impact. For instance, comparable and uniform enhancements in absorption capacity were observed for yellow, red, and blue dyes, as well as their mixtures, on the substrate as the dyeing pressure increased from 15 to 25 MPa. When all other parameters are constant, pressure has a direct impact on how the dye particles dissolve in the dyeing medium. A higher concentration of dye particles dissolves and remains in the supercritical bulk fluid at higher pressures throughout the dyeing process. This boost results in enhancing the RD dye absorption characteristics by facilitating better dye molecule transfer, thermal diffusion, and adsorption on wool fibers^54,55^.

#### The effect of time on dyed wool samples

**Fig. 5:**
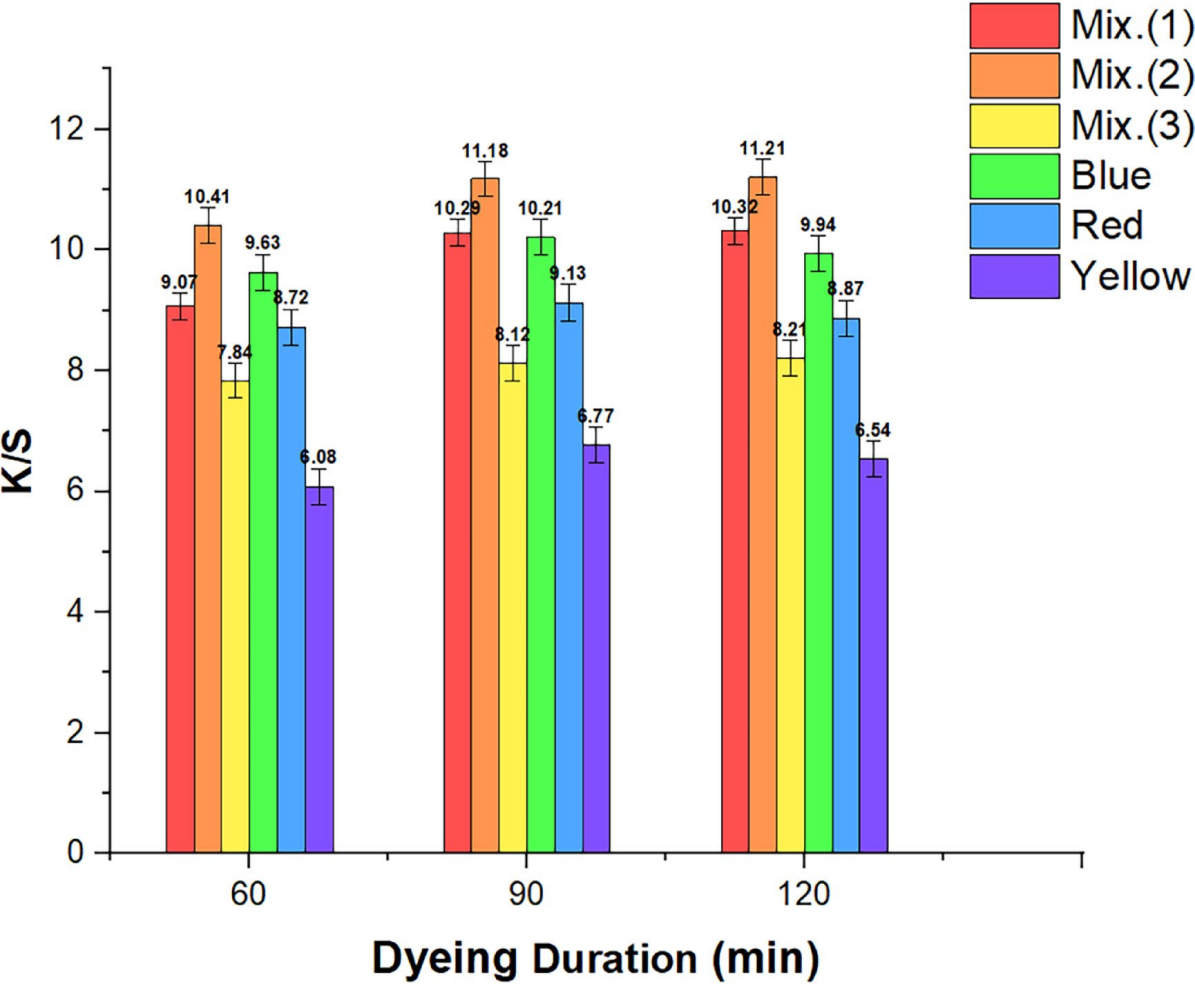
Relationship graphs for the three dyes under investigation and their 50:50 combination between dyeing duration and *K/S*. (Mix 1 symbolizes the mixture of yellow and blue dyes, Mix 2 symbolizes the mixture of red and blue dyes, and Mix 3 symbolizes the mixture of yellow, red, and blue dyes).

Dyeing time is critical in the SCDD and color-matching process. As a result, the investigation focused on determining the impact of dyeing time on absorption behavior and the compatibility of RD dyes under constant pressure and temperature conditions. Based on the outcomes in Fig. 5, the *K/S* readings for the wool material improved once the dyeing duration was expanded from 60 to 90 min. The considerable rise in value led to the preference for 90 min as the ideal period for further exploration of other factors. The choice to concentrate on the 90-min mark was established by finding that more reactive dye groups linked to fiber structures during this time frame, with negligible additional absorption at 120 min.

#### Compatibility evaluation of dyed samples

Table 2 presents the colorimetric data for dyed wool samples compared to the blank wool for the different mixtures of yellow, red, and blue dye. The hue shift (∆H), chroma variation (∆C), metamerism index (MI), and overall color difference (∆E) are among the data that are presented.

**Table 2:** Color parameters of wool fabrics dyed with a mixture of yellow, red, and blue reactive disperse dyes.

Dyes- combination	*K/S*	∆E	∆C	∆H	MI	CDI
The blends of Y dye and B dye						
M1 (B dye: Y dye 80:20)	11.14	25.5	4.5	2.6	0.32	4.7
M2 (B dye: Y dye 75:25)	10.91	22.5	7.1	3.5	0.43	4.7
M3 (B dye: Y dye 50:50)	10.29	29.4	18.22	5.6	0.51	4.6
M4 (B dye: Y dye 25:75)	9.84	21.23	11.5	8.9	0.23	3.7
M5 (B dye: Y dye 20:80)	8.91	24.37	12.2	5.5	0.42	4.6
The blends of R dye and B dye						
M1 (B dye: R dye 80:20)	11.9	26.26	9.6	7.1	0.21	4.07
M2 (B dye: R dye 75:25)	11.41	23.40	18.5	10.11	0.32	4.09
M3 (B dye: R dye 50:50)	11.18	24.71	20.37	8.6	0.41	4.2
M4 (B dye: R dye 25:75)	9.86	33.59	11.6	4.5	0.33	4.3
M5 (B dye: R dye 20:80)	9.11	25.62	11.22	7.4	0.25	4.22
The blends of B dye , R dye and Y dye						
M1 (B dye: R dye: Y dye (2:1:1)	8.87	28.22	16.6	10.2	0.28	4.85
M2 (B dye: R dye: Y dye 1:2:1)	8.63	31.48	19.99	7.13	0.43	4.83
M3 (B dye: R dye: Y dye 1:1:1)	8.57	22.33	18.3	8.1	0.49	4.84
M4 (B dye: R dye: Y dye 1:1:2)	8.43	32.1	16.3	4.7	0.52	4.81

The Color Difference Index (CDI) Table 3 was utilized in this study to further clarify the relationship between color accumulation rate, chroma, metamerism, hue, and the percentage/fluctuation of dyeing process factors on the overall amount of color differences. The CDI provides a consolidated metric that captures the cumulative influence of several color difference characteristics when dyeing diverse shades under SCDD. Hence, elevated CDI values signal the necessity for meticulous control of color variation within a specific dyeing scenario and fine-tuning individual dye ratios in a multi-component shade formulation to create intricate color palettes.

**Table 3:** CDI and compatibility grade of mixed dyed wool samples.

Binary pair of dye combinations						CDI _max_ − CDI _min_ difference	RCR	Compatibility grade as per RCR values
	80:20	75:25	50:50	25:75	20:80			
B dye: Y dye	4.7	4.7	4.6	3.7	4.6	4.7–4.6 = 0.1	4	Good
B dye: R dye	4.07	4.09	4.2	4.3	4.22	4.3–4.07 = 0.23	3–4	Moderate

Binary pair of dye combinations	CDI values					CDI _max_ − CDI _min_ difference	RCR	Compatibility grade as per RCR values
	2:1:1	1:2:1	1:1:1	1:1:2	–			
B dye: R dye: Y dye	4.85	4.83	4.84	4.81	–	4.85–4.81 = 0.04	4–5	Excellent

To be more precise, the dyeing procedure employed a single RD dye, which was then mixed under study. It maintained the same dyeing conditions and shade depths for every sample.

#### Color performance test of dyed samples

**Fig. 6:**
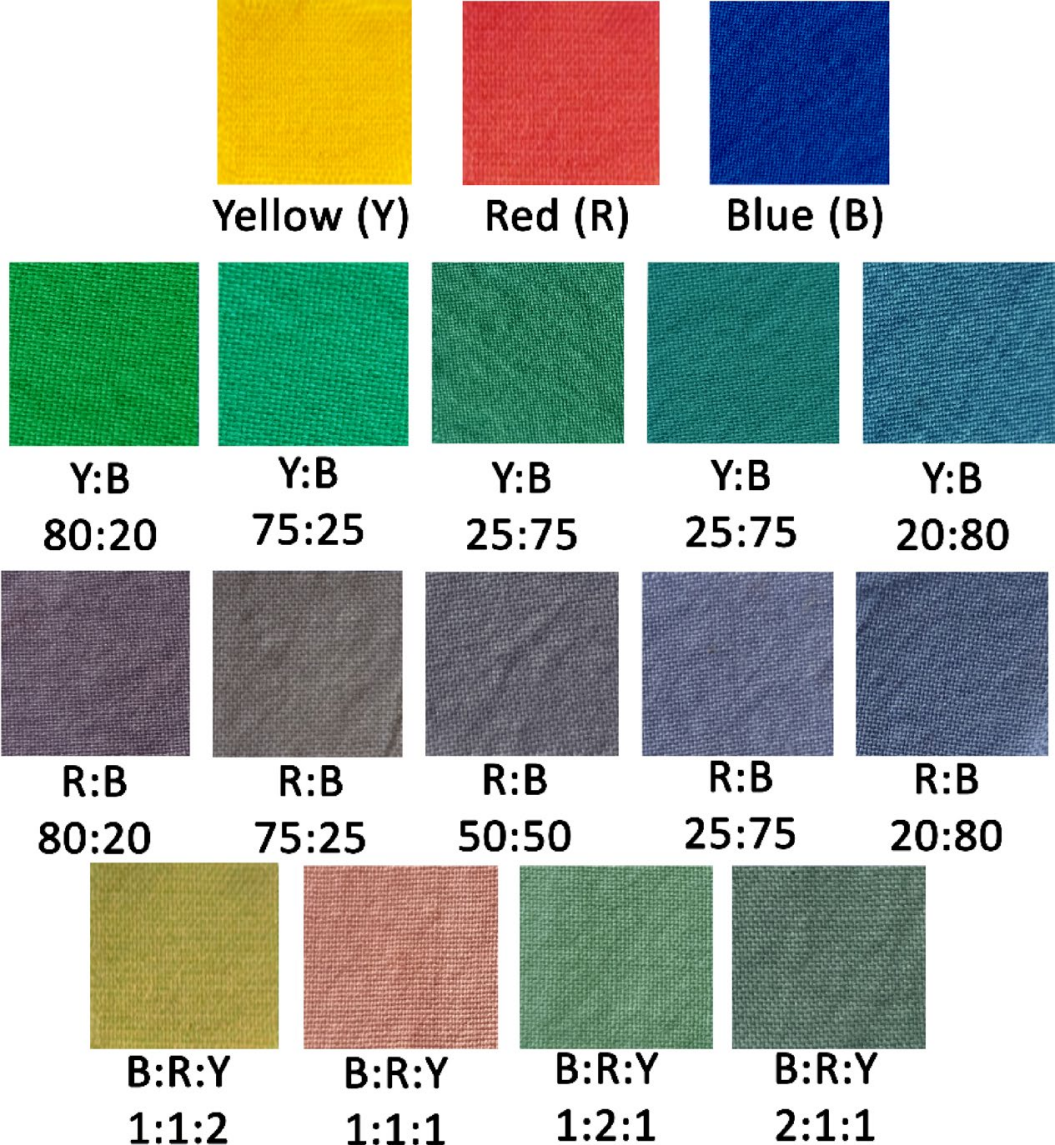
Digital image of dyed materials with the mixture of dyes.

L* = 0 and L* = 100, respectively, stand for pure black and white. Regarding the blue sample, the color lightness value (L*) reported was 26.22, which shifted towards darkness, as demonstrated in Table 4**.** However, the yellow sample shifted towards whiteness, with a value of 90.02, and the red samples shifted towards 49.5. The L* values of the mixed dyed wool samples ranged from 36.14 to 55.16. Negative a* values show that the color tones of the blended blue and yellow dye samples have a greenish inclination along the red-green axis. In a similar vein, the color tones of most blended samples are shifted toward the bluish end of the yellow-blue axis on wool fabrics due to negative b* values. Nonetheless, samples M3, M4, and M5 have positive b* values, reflecting a greater concentration of yellow hues in these instances as shown in Fig. 6. Except samples M3, M4, and M5, the negative values of a* cause the color tones of the blended blue and red dye to shift slightly in a greenish orientation on the red-green axis. Except for M5, the color of the wool fabrics altered to a bluish orientation along the yellow-blue axis when b* was negative. The samples with values on the a* and b* axes near (0,0), indicating gray shades, were observed. In contrast, samples with values of a* near 0 and b* trending towards the blue direction exhibited grayish blue tones. While the mixture of the three dyes showed different tones of colors with different shifted values of a* and b*, as shown in Fig. 6 and Table 4. M1 indicated a very slight toward teal or blue-green tone. For M2, increasing the red dye ratio shifted the color tone slightly towards green tones, while M3 exhibited a light, warm reddish-brown. By increasing the yellow dye ratio for M4, an olive tone was observed. With different shifted values of a* and b* as indicated in Fig. 6 and Table 4. In terms of color purity (C*) values, the degree of intensity, richness, vigor, or purity of a color is correlated with its saturation. Another word frequently used to refer to this dimension is chroma; despite their technical differences, these terms are similar. It is essential to note that different colors have varying maximum saturations; that is, some colors have higher upper limit saturations than others. A color’s appearance grows stronger or purer as its saturation (chroma) rises, and it starts to appear washed out or pale as its saturation falls. The chroma of the mixed blue dye and yellow dye samples showed higher values than the mixed blue dye and red dye samples. h° represents the hue angle (0° (red)—60° (yellow)—120° (green)—180° (cyan)—240° (blue)—300° (magenta)—360° (red)), mainly employed to distinguish between various colors. The h° values of the blended dyed samples varied from 90.43 to 280.19.

**Table 4:** Color analysis of dyed wool materials.

	Dyes- combination	L*	a*	B	CV % OF K/S	Stdv	Standard Error	K/S	C*	H
1	B Dyed sample	**26.22**	**9.40**	**− 28.99**	**0.62**	**0.06**	**0.035**	**9.87**	**31.60**	**286.71**
2	Y Dyed sample	**90.02**	**− 7.98**	**− 71.45**	**0.430**	**0.032**	**0.018**	**7.5**	**68.5**	**85.6**
3	R Dyed sample	**49.5**	**49.7**	**22.4**	**0.71**	**0.06**	**0.035**	**8.55**	**37.5**	**25.15**
The blends of B dye and Y dye	M1 (B dye: Y dye 80:20)	46.57	− 15.3	− 11.27	0.73	0.081	0.047	11.14	16.21	214.87
	M2 (B dye: Y dye 75:25)	50.34	− 18.69	− 18.75	3.00	0.31	0.183	10.91	24.5	110.11
	M3 (B dye: Y dye 50:50)	44.33	− 18.38	2.35	1.44	0.146	0.084	10.29	18.53	172.72
	M4 (B dye: Y dye 25:75)	55.16	− 17.8	12.72	2.7	0.258	0.149	9.84	21.87	144.46
	M5 (B dye: Y dye 20:80)	52.45	− 17.54	5.58	3.69	0.315	0.182	8.91	18.4	162.35
The blends of R dye and B dye	M1 (B dye: R dye 80:20)	49.99	− 2.06	− 9.87	2.15	0.25	0.144	11.9	10.08	258.2
	M2 (B dye: R dye 75:25)	52.88	− 0.71	− 8.75	2.34	0.26	0.151	11.41	6.78	264.84
	M3 (Be dye: R dye 50:50)	52.41	0.7	− 3.88	0.96	0.10	0.061	11.18	3.94	280.19
	M4 (B dye: R dye 25:75)	36.14	2.78	− 1.28	1.57	0.15	0.08	9.6	11.22	199.2
	M5 (B dye: R dye 20:80)	39.2	2.08	0.08	1.6	0.14	0.08	9.11	2.09	210.1
The blends of B dye, R dye and Y dye	M1 (B dye: R dye: Y dye 2:1:1)	48.84	− 3.28	− 1.61	1.81	0.158	0.09	8.87	3.65	206.2
	M2 (B dye: R dye: Y dye 1:2:1)	50.85	− 4.72	1.36	0.61	0.052	0.03	8.63	4.91	163.97
	M3 (B dye: R dye: Y dye 1:1:1)	38.84	5.82	4.08	0.82	0.07	0.04	8.57	35.02	100.56
	M4 (B dye: R dye: Y dye 1:1:2)	47.23	− 0.07	9.66	0.72	0.06	0.03	8.43	9.66	90.43

### Leveling and fixation characteristics of dyed fabrics

To assess color variation in colored wool fabric, leveling features were measured at five distinct spots. Measurements were taken at concentrations ranging from 1 to 6% (o.w.f.) at each location. Table 5 shows that all dyed wool fabrics with varying dye concentrations had ΔE values below 1. Wool materials showed good leveling properties after SCCO_2_ dyeing. A dye concentration of 4% exhibits higher leveling properties than other dye concentrations.

**Table 5:** Leveling characteristics of dyed wool samples.

	Mix ratio	1%	2%	3%	4%	6%
The blends of B dye and Y dye	∆E _80:20_	0.95	0.86	0.56	0.44	0.72
	∆E_75:25_	1.1	0.99	0.67	0.81	0.55
	∆E_50:50_	0.77	0.74	0.44	0.64	0.81
	∆E_25:75_	0.83	0.78	0.71	0.74	0.76
	∆E_20:80_	0.79	0.66	0.48	0.36	0.65
The blends of B dye and R dye	∆E _80:20_	1.5	1.1	0.78	0.55	0.87
	∆E_75:25_	1.3	0.89	0.68	0.34	0.56
	∆E_50:50_	0.99	0.67	0.97	0.26	0.34
	∆E_25:75_	0.87	0.99	0.58	0.71	0.54
	∆E_20:80_	0.65	1.1	0.26	0.34	0.88
The blends of B dye , R dye and Y dye	∆E_2:1:1_	0.79	0.45	0.67	0.45	0.26
	∆E_1:2:1_	0.87	0.65	0.78	0.78	0.59
	∆E_1:1:1_	0.78	0.47	0.68	0.56	0.19
	∆E_1:1:2_	0.99	0.55	0.88	0.33	0.56

Table 6 shows that varying combinations of two and three dyes under examination resulted in high fixations, ranging from 76.02 to 90.8, on wool fibers.

**Table 6:** Fixation percentage of dyed wool samples.

	Blended samples	*K/S* after dyeing	*K/S* after extraction	Fixation (%)
The blends of B dye and Y dye	M1 (B dye: Y dye 80:20)	11.14	9.88	88.68
	M2 (B dye: Y dye 75:25)	10.91	9.23	84.6
	M3 (B dye: Y dye 50:50)	10.29	8.22	79.88
	M4 (B dye: Y dye 25:75)	9.84	8.9	90.8
	M5 (B dye: R dye 20:80)	8.91	7.78	87.3
The blends of B dye and R dye	M1 (B dye: R dye 80:20)	11.9	10.55	88.65
	M2 (B dye: R dye 75:25)	11.41	9.4	82.38
	M3 (B dye: R dye 50:50)	11.18	8.5	76.02
	M4 (B dye: R dye 25:75)	9.86	7.99	81.03
	M5 (B dye: R dye 20:80)	9.11	8.34	91.54
The blends of B dye, R dye and Y dye	M1 (B dye: R dye: Y dye 2:1:1)	9.04	7.89	87.27
	M2 (B dye: R dye: Y dye 1:2:1)	8.84	7.22	81.67
	M3 (B dye: R dye: Y dye 1:1:1)	8.12	7.21	88.79
	M4 (B dye: R dye: Y dye 1:1:2)	8.48	6.99	82.42

B–Y refers to the mixture of blue and yellow dyes, B–R refers to the mix of blue and red dyes, and B–R–Y refers to the mixture of the three dyes.

The dye molecules theoretically form a linkage with the base fibers throughout the SCDD procedure, employing a nucleophilic substitution reaction with the functional groups on the macrochains of the base fabrics.

### Color fastness of dyed samples

Employing the dyes and their blends within the investigation that were obtained under SCCO_2_ at (pressure: 25 MPa, dye concentration: 4% o.m.f., temperature: 110 °C, and dyeing duration: 90 min.), all experiments were conducted under optimal conditions. The proposed dyes demonstrated exceptional staining and fading washing durability, scoring a 5 in the international grayscale, as displayed in Table 7. Thanks to the dyes’ efficient diffusion and penetration. The dyed wool fabrics exhibited remarkable resistance to sweat and (wet & dry) rubbing, establishing a grade of 5. Nonetheless, the lightfastness findings were favorable, with values ranging from 4 to 5. Following ten cycles of washing durability testing, positive results were observed. The results were achieved as the dye mixture reacted significantly with the wool fibers, likely resulting in the formation of novel chemical interactions between the wool and the dyes.

**Table 7:** Fastness characteristics associated with SCCD-dyed wool specimens.

	Dye mixture	Wash fastness		Crocking fastness		Perspiration fastness				Light fastness
		Shade	Stain	Wet	Dry	Acidic		Alkali		
						Shade	Stain	Shade	Stain	
The blends of B dye and Y dye B:Y	Blue Dye	5 (4)	5	5	5	5	5	5	5	4–5
	Red Dye	5(4)	5	5	5	5	5	5	5	4–5
	Yellow Dye	5 (4)	5	5	5	5	5	5	5	4–5
	M1 (80:20)	5 (4)	5	4–5	5	5	5	5	5	4–5
	M2 (75:25)	5 (4)	5	5	5	5	5	5	5	4–5
	M3 (50:50)	5 (4)	5	5	5	5	5	5	5	4–5
	M4 (25:75)	5 (4)	5	5	5	5	5	5	5	4–5
	M5 (20:80)	5 (4)	5	5	5	5	5	5	5	4–5
The blends of B dye and R dye B:R	M1 (80:20)	5 (4)	5	5	5	5	5	5	5	4–5
	M2 (75:25)	5 (4)	5	4–5	5	5	5	5	5	4–5
	M3 (50:50)	5(3–4)	5	5	5	5	5	5	5	4–5
	M4 (25:75)	5 (4)	5	5	5	5	5	5	5	4–5
	M5 (20:80)	5(3–4)	5	5	5	5	5	5	5	4–5
The blends of B dye , R dye and Y dye B:R:Y	M1 (2:1:1)	5 (4)	5	4–5	5	5	5	5	5	4–5
	M2 (1:2:1)	5 (4)	5	5	5	5	5	5	5	4–5
	M3 (1:1:1)	5 (4)	5	5	5	5	5	5	5	4–5
	M4 (1:1:2)	5 (4)	5	5	5	5	5	5	5	4–5

The values enclosed in parentheses represent the washing durability test.

## Conclusion

The current research efficiently established the feasibility of SCDD of wool fabric using reactive disperse dyes. The systematic investigation of dye concentration, dye blends, pressure, temperature, and dyeing time demonstrated their significant impact on color strength, fixation quality, and leveling features. A color uniformity test showed that the RD dyes were uniformly distributed in the wool yarns when using binary and ternary dye mixtures, resulting in a consistent color. Furthermore, the dyed samples exhibited satisfactory fastness performance, with minimal shade loss upon durability washing. Collectively, our findings overcome the limits of traditional aqueous wool dyeing by introducing a sustainable approach that minimizes water and chemical usage while widening the color spectrum. The findings support the advancement of SCCO_2_ technology as a feasible and environmentally responsible option for wool dyeing in future textile applications.

## Data Availability

All data generated or analysed during this study are included in this published article.

## Abbreviations

SCCO_2_: Supercritical carbon dioxide
SCDD: Supercritical carbon dioxide dyeing
RD: Reactive disperse
B dye: Blue dye
R dye: Red dye
Y dye: Yellow dye
CDI: Color difference index
